# Different Expression Levels of Human Mutant Ubiquitin B^+1^ (UBB^+1^) Can Modify Chronological Lifespan or Stress Resistance of *Saccharomyces cerevisiae*

**DOI:** 10.3389/fnmol.2018.00200

**Published:** 2018-06-08

**Authors:** Ana Joyce Muñoz-Arellano, Xin Chen, Andrea Molt, Eugenio Meza, Dina Petranovic

**Affiliations:** ^1^Division of Systems and Synthetic Biology, Department of Biology and Biological Engineering, Chalmers University of Technology, Gothenburg, Sweden; ^2^Novo Nordisk Foundation Center for Biosustainability, Chalmers University of Technology, Gothenburg, Sweden

**Keywords:** UBB^+1^, proteasome, Alzheimer’s disease, aging, cell death, yeast

## Abstract

The ubiquitin-proteasome system (UPS) is the main pathway responsible for the degradation of misfolded proteins, and its dysregulation has been implicated in several neurodegenerative diseases, including Alzheimer’s disease (AD). UBB^+1^, a mutant variant of ubiquitin B, was found to accumulate in neurons of AD patients and it has been linked to UPS dysfunction and neuronal death. Using the yeast *Saccharomyces cerevisiae* as a model system, we constitutively expressed UBB^+1^ to evaluate its effects on proteasome function and cell death, particularly under conditions of chronological aging. We showed that the expression of UBB^+1^ caused inhibition of the three proteasomal proteolytic activities (caspase-like (β1), trypsin-like (β2) and chymotrypsin-like (β5) activities) in yeast. Interestingly, this inhibition did not alter cell viability of growing cells. Moreover, we showed that cells expressing UBB^+1^ at lower level displayed an increased capacity to degrade induced misfolded proteins. When we evaluated cells during chronological aging, UBB^+1^ expression at lower level, prevented cells to accumulate reactive oxygen species (ROS) and avert apoptosis, dramatically increasing yeast life span. Since proteasome inhibition by UBB^+1^ has previously been shown to induce chaperone expression and thus protect against stress, we evaluated our UBB^+1^ model under heat shock and oxidative stress. Higher expression of UBB^+1^ caused thermotolerance in yeast due to induction of chaperones, which occurred to a lesser extent at lower expression level of UBB^+1^ (where we observed the phenotype of extended life span). Altering UPS capacity by differential expression of UBB^+1^ protects cells against several stresses during chronological aging. This system can be valuable to study the effects of UBB^+1^ on misfolded proteins involved in neurodegeneration and aging.

## Introduction

The ubiquitin proteasome system (UPS) is the principal proteolytic pathway that degrades proteins in a regulated manner. Its substrates are targeted for degradation by ubiquitin, a 76-amino-acid polypeptide that is covalently attached to one or more lysine residues of cellular proteins in a process called ubiquitylation. Protein ubiquitylation is mediated by an enzymatic cascade consisting of ubiquitin activating (E1), conjugating (E2) and ligating (E3) enzymes which generates a polyubiquitin chain that functions as the degradation signal. The ubiquitin-conjugated proteins are subsequently recognized and degraded by the 26S proteasome to short peptides, while reusable ubiquitin is released by deubiquitinating enzymes (DUBs; Welchman et al., 2005).

The UPS is involved in a variety of cellular functions such as intracellular signaling, regulation of cell cycle and induction of apoptosis (Hershko and Ciechanover, 1998). Deficiencies in either ubiquitin or proteasome, unless timely corrected, can cause cellular dysfunction or death. An example that ubiquitin itself can be a cause of UPS impairment is the aberrant ubiquitin UBB^+1^. This mutant ubiquitin results from a dinucleotide “deletion” probably occurring during transcription near the 3’ mRNA end, at the first of three ubiquitin repeats of ubiquitin B gene *UBB* (van Leeuwen et al., 1998a). This process, termed molecular misreading (van Leeuwen et al., 2000), preferentially occurs at sequence repeats like GAGAG with 1:10^5^ frequency in different cell types. The aberrant mRNA results in the translation of UBB^+1^, which has a 20 amino acid C-terminal extension, with the rest of the protein being identical to ubiquitin (van Leeuwen et al., 1998b). Due to this modification, the C-terminal glycine, which is essential for the role in conjugation of substrates, is missing. Thus, UBB^+1^ cannot be conjugated to targets, but itself is ubiquitylated at lysines 29 and 48 and further degraded by the proteasome (Lam et al., 2000; Lindsten et al., 2002). Although UBB^+1^ is successfully degraded under normal conditions when its levels are very low in the cells (Lindsten et al., 2002), it tends to accumulate specifically in affected cells in several neurodegenerative diseases characterized by the presence of misfolded proteins, such as Alzheimer’s disease (AD) and Huntington’s disease (van Leeuwen et al., 1998b; de Pril et al., 2004), as well as in some non-neuronal pathologies such as liver pathologies (French et al., 2001; Wu et al., 2002) and sporadic inclusion body myositis muscle fibers (Fratta et al., 2004). Shabek et al. showed that the polyubiquitin chain on UBB^+1^ increases its affinity for the proteasome, and because UBB^+1^ cannot be efficiently degraded, it inhibits degradation of other ubiquitin-dependent proteasome substrates (Shabek et al., 2009). Moreover Krutauz et al. demonstrated that extended ubiquitin variants affect the ubiquitin-dependent turnover by inhibiting deubiquitination activity of proteasome associated DUBs (Krutauz et al., 2014). Thus, when UBB^+1^ accumulates to higher levels, under conditions where the capacity of UPS is challenged, this can result in a positive feedback loop in which the already compromised UPS is further inhibited due to the accumulated UBB^+1^ (Verhoef et al., 2009). Hence, the presence of UBB^+1^ in human tissue serves as a reporter for UPS impairment (Fischer et al., 2003). Since elevated levels of UBB^+1^ inhibit the UPS (Lindsten et al., 2002; Hope et al., 2003), cells can be driven to cell cycle arrest, neuritic beading, mitochondrial stress and apoptosis (De Vrij et al., 2001; Lindsten et al., 2002; Hope et al., 2003; Tan et al., 2007). Studies in HeLa cells and cortex slice cultures expressing UBB^+1^ showed that there is a dose-dependent shift in UBB^+1^ properties from UPS substrate to UPS inhibitor with increasing expression levels (van Tijn et al., 2007). In human neuroblastoma cells, induction of UBB^+1^ causes proteasome inhibition as well as induction of heat shock proteins (HSPs), the latter presumably promoting the observed resistance to oxidative stress (Hope et al., 2003). Hence, in this model the protective effect of HSPs is bigger than the deleterious inhibition of proteasome by UBB^+1^.

UBB^+1^ transgenic mouse lines have been established with post-natal neuronal expression of UBB^+1^ (Fischer et al., 2009). These mice showed increased levels of ubiquitylated proteins in the cortex, at least 20% decreased proteasome activity, expression changes in proteins similar to the proteomic profiles of AD brain as well as the deficit in contextual memory. The same mouse line has been used in a study where in addition to UBB^+1^ also expanded huntingtin constructs were expressed using a lentiviral system (de Pril et al., 2010). This resulted in increased neuronal inclusion formation demonstrating that UBB^+1^ transgenic mice are more vulnerable to toxic protein accumulation. Similar results were observed by Tank and True expressing a protein analogous to UBB^+1^, Ub^ext^, in baker’s yeast. They found out that UBB^+1^ causes UPS impairment and makes cells more susceptible to toxic protein aggregates (Tank and True, 2009).

Although UBB^+1^ has been studied in several *in vivo* models, the exact molecular mechanism for UBB^+1^-mediated UPS dysfunction and its contribution to cell death in AD is still unclear. Using a yeast model, Braun et al. (2015a,b) showed that UBB^+1^ expressed from a high-copy vector under the control of an inducible promoter leads to UPS impairment, mitochondrial dysfunction and toxic impairment of the basic amino acids synthesis. Yeast *S. cerevisiae* is a suitable model for aging and cell death studies, including apoptosis and necrosis, as well as for the study of protein quality control mechanisms, which are key cellular processes involved in neurodegeneration and are well conserved between yeast and higher eukaryotes (Khurana and Lindquist, 2010). In this study, we constitutively expressed UBB^+1^ at lower and higher expression levels under different conditions, including those of chronological aging. We aimed at modeling conditions that would occur naturally in aging neurons and evaluated the effects on proteasome function and cell death. We showed that UBB^+1^ inhibited all three types of proteolytic subunits of the proteasome. However the constitutive expression of low level of UBB^+1^ increased cellular resistance to misfolded proteins and moreover prevented cells from accumulating reactive oxygen species (ROS) during aging, delayed cell death and increased chronological life span (CLS). On the other hand high UBB^+1^ expression besides causing proteasomal inhibition, conferred resistance to heat shock stress in exponential phase, and this did not affect cell survival under aging conditions.

## Materials and Methods

### Plasmids and Strains

**Figure 1:**
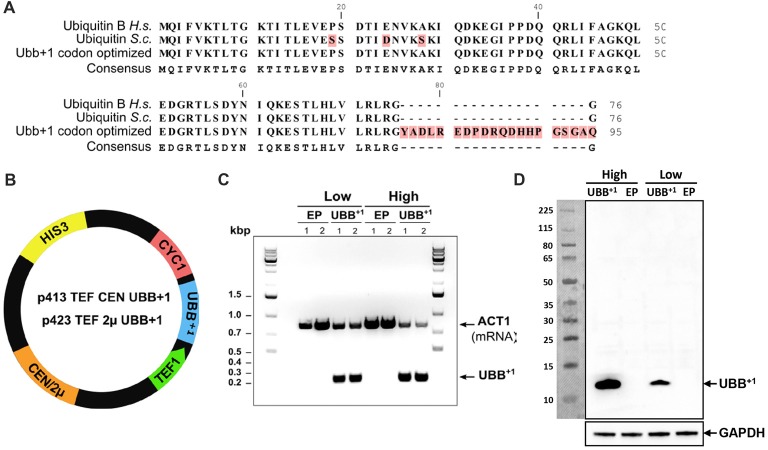
Human UBB^+1^ expression in yeast *S. cerevisiae*. **(A)** Alignment of ubiquitin protein sequences from human (Ubiquitin B *H.s*.), yeast (Ubiquitin *S.c*.) and *UBB*^+1^ codon optimized for expression in yeast. The UBB^+1^ protein sequence is depicted with the 20 amino acid C-terminal extension. **(B)** Schematic map of the *UBB*^+1^ expression plasmids. *HIS3* stands for histidine auxotrophic marker, *CEN* or 2μ is the origin of replication, *TEF1* is the promoter sequence and* CYC1* is the terminator sequence. **(C)** RT-PCR verifies the transcription of *UBB*^+1^ in Low and High_UBB^+1^ strains. The controls carried empty plasmid (EP) without *UBB*^+1^ gene. Housekeeping gene *ACT1* was used as a reference gene. **(D)** Western blot analysis of UBB^+1^ expression from exponential growth cells, using the anti-ub^+1^ antibody. Expression of UBB^+1^ (12 kDa) is indicated by an arrow.

Plasmids and strains used and constructed in this study are listed in Table 1. Plasmid p413TEF CEN (Mumberg et al., 1995) is a centromeric plasmid (1–2 copies) that carries the marker genes *HIS3* and *ampC*, for maintenance and propagation in *S. cerevisiae* and *Escherichia coli*, respectively (Figure 1B). This plasmid has a multicloning site flanked by the constitutive promoter of translation elongation factor 1α (TEF1) and cytochrome c terminator (CYC1). The p423 TEF 2μ plasmid (Figure 1B) shares the same features as p413 TEF CEN but it is a high copy number plasmid, due to its episomal autonomous origin of replication (Mumberg et al., 1995). To create the p413TEF UBB^+1^ and p423TEF UBB^+1^ plasmids, human *UBB*^+1^ gene was synthesized by DNA2.0 Inc. with a yeast Kozak sequence (Hamilton et al., 1987) at the 5’ end, and the *UBB*^+1^ codons were optimized for efficient expression in *S. cerevisiae*. The synthetic gene coding was inserted into pJ204 plasmid resulting in pJ204:28004-UBB^+1^. Then UBB^+1^ was subcloned from pJ204:28004-UBB^+1^ into p413TEF CEN and p423TEF 2μ plasmids. Constructed plasmids were purified and verified by restriction analysis and sequencing (GATC Biotech AB, Sweden).

**Table 1:** Plasmids and strains used in this study.

Plasmids or strains	Relevant genotype	Reference
**Plasmids**		
pJ204:28004-UBB^+1^	*UBB*^+1^ Amp^R^ (*bla*)	This work
p413TEF CEN	CEN *HIS3* P_*TEF*1_/T_*CYC*1_ Amp^R^ (*bla*)	Mumberg et al. (1995)
p423TEF 2μ	2μ *HIS3* P_*TEF*1_/T_*CYC*1_ Amp^R^ (*bla*)	Mumberg et al. (1995)
p413TEF CEN UBB^+1^	CEN *HIS3* P_*TEF*1_-*UBB*^+1^-T_*CYC*1_ Amp^R^ (*bla*)	This work
p423TEF 2μ UBB^+1^	2μ *HIS3* P_*TEF*1_-*UBB*^+1^-T_*CYC*1_ Amp^R^ (*bla*)	This work
***S. cerevisiae* strains**		
CEN.PK113-11C	MATa *his*3Δ1 *ura 3-52* MAL-8c SUC2	van Dijken et al. (2000)
Low_EP	CEN.PK113-11C, p413TEF CEN	This work
Low_UBB^+1^	CEN.PK113-11C, p413TEF CEN UBB^+1^	This work
High_EP	CEN.PK113-11C, p423TEF 2μ	This work
High_UBB^+1^	CEN.PK113-11C, p423TEF 2μ UBB^+1^	This work
*atg1*Δ	CEN.PK113-11C Δ*Atg1*	This work
*atg1*Δ_Low_UBB^+1^	CEN.PK113-11C Δ*Atg1*, p413TEF CEN UBB^+1^	This work
***Escherichia coli***		
DH5α	*fhuA2*Δ (*argF-lacZ*) *U169 phoA glnV44* Φ80Δ (*lacZ*)*M15*	Bryant (1988)
	*gyrA96 recA1 relA1 endA1 thi-1 hsdR17*

*Eshcerichia coli* DH5α strain (*fhuA2*Δ (*argF-lacZ*) *U169 phoA glnV44* Φ80Δ (*lacZ*)*M15 gyrA96 recA1 relA1 endA1 thi-1 hsdR17*; Inoue et al., 1990) was used for cloning. The *S. cerevisiae* strains used in this study were derivatives of haploid CEN.PK113-11C (*MATa his3Δ*1 *ura 3-5*2 *MAL-8c SUC2*).* S. cerevisiae* CEN.PK 113-11C was transformed with purified p413TEF CEN, p423TEF 2μ, p413TEF UBB^+1^ and p423TEF UBB^+1^ by standard procedures (Gietz and Woods, 2002) to generate the strains Low_EP, High_EP, Low_UBB^+1^ and High_UBB^+1^, respectively (Table 1). Transformants were selected on synthetic minimal medium lacking histidine (SD-His^−^. The presence of *UBB*^+1^ gene in the resulting yeast strains was verified by PCR (Fisher Scientific, Sweden).

For *Atg1* deletion, the knockout cassette was constructed with a PCR amplified KanMX marker (from pUG6 plasmid) including approximately 500 bp upstream and downstream of the *Atg1* locus. The CEN.PK 113-11C strain was transformed with this cassette. Correct integration in the genome was confirmed by PCR (unpublished data).

### Cultivation Medium and Growth Conditions

Unless otherwise indicated, strains were grown in synthetic drop-out medium containing yeast nitrogen base without aminoacids (Formedium, Hunstanton, England) and a complete supplement amino acid mixture lacking histidine (SD-His^−^; Formedium, England), with 2% glucose as carbon source. Strains were grown on SD-His^−^ plates for 2–3 days at 30°C or in liquid SD-His^−^ medium (either in tube or shake flasks with a 1:5 medium/volume flask ratio) at 30°C with 200 rpm shaking.

Initial innoculation of the liquid SD- His^−^ medium was at OD_600_ of 0.15. The growth was monitored by measuring OD_600_ every 90 min during the first 12 h and at different time points of stationary phase.

All experiments were performed with samples from at least two independent transformants, and in triplicate, unless specified.

### Verification of *UBB^+1^* Gene Transcription by RT-PCR

Strains Low_EP, High_EP, Low_UBB^+1^ and High_UBB^+1^ were grown in SD- His^−^ medium overnight. At OD_600_ ~2.5, cells were harvested and total RNA was extracted with the waterbath method (Li et al., 2009). After verifying concentration and quality of RNA samples, 1 μg of each RNA sample was treated with RNase-free DNase I (Qiagen, Sweden) according to the manufacturer’s instructions. First-strand cDNA synthesis was performed with 1 μg of DNase treated RNA using random hexamers and RevertAid H Minus M-MuLV Reverse Transcriptase (Thermo Fisher Scientific, Sweden) according to the manufacturer’s instructions. Subsequently, multiplex PCR was carried out for *UBB^+1^* and *ACT1* genes using Phusion High-Fidelity DNA polymerase (Thermo Fisher Scientific, Sweden). Housekeeping gene *ACT1* was used as a reference gene to normalize RNA levels.

### Verification of UBB^+1^ Translation by Western Blot

Protein extraction for exponential phase cells was performed as described previously (Chen and Petranovic, 2015). Five OD_600_ of cells were spun down and resuspended in 200 μl of lysis buffer containing 50 mM HEPES (pH 7.5), 150 mM NaCl, 2.5 mM EDTA, 1% Triton X-100 with Complete Mini Protease Inhibitor (Roche, Switzerland). Two-hundred microliter of glass beads (MP Biomedicals, USA) was added to the lysis buffer, then the cells were mechanically disrupted at 4°C for 3 min on the FastPrep homogenizer (MP Biomedicals, USA). Afterwards, samples were centrifuged and the supernatant was collected as lysate. BCA protein assay kit (Thermo Scientific, USA) was used to measure protein concentrations in the lysate and 30 μg of protein for each sample was loaded on a 4%–12% Bis-Tris gel (Invitrogen, USA).

Protein extraction from stationary phase cells was done according to Kushnirov (Kushnirov, 2000), in 50 μl reducing ClearPAGE™ LDS Sample Buffer (VWR international AB, Sweden). Samples were heated for 10 min at 70°C. About 10 μl of protein extract (0.6 OD_600_) was loaded per lane of 4%–12% Bis-Tris gel (Invitrogen, USA). After electrophoresis, proteins were transferred onto 0.2 micron PVDF membrane (BioRad, USA) using a semi-dry transfer system (Towbin et al., 1979; Kyhse-Andersen, 1984). Monoclonal antibodies against UBB^+1^ (Ub^+1^ 40B3, Santa Cruz Biotechnology, Sweden) and GAPDH (Santa Cruz Biotechnology, Sweden) were used for Western blot. Enhanced chemiluminescence signals were detected using the ECL Prime reagent (GE Healthcare, USA) and the ChemiDoc XRS imaging system (BioRad, USA).

### Proteasome Activity Measurements

Luminescence activity measurements of the chymotrypsin-, caspase- and trypsin-like proteolytic activities of 20S proteasome were performed using the Proteasome-Glo™ Cell-Based Assay from Promega according to the manufacturer’s instructions (Promega Biotech, Sweden). The Proteasome-GloTM Cell-Based Reagents each contains a specific luminogenic proteasome substrate in a buffer optimized for cell permeabilization, proteasome activity and luciferase activity. The specific proteasome can recognize and cleave luminogenic substrates, resulting in a luminescent signal that is proportional to the amount of proteasome activity in cultured cells. One-hundred microliter of SD-His^−^ medium containing defined numbers of cells (20,000, 40,000 and 80,000) was mixed with 100 μl of Cell-Based Reagent and incubated for 20 min at room temperature. Luminescence was recorded using the FLUOstar Galaxy plate reader from BMG Labtechnologies (Sweden). Every sample was measured in duplicate on two independent transformants and in three independent experiments.

In order to exclude that the detected signal resulted from non-proteasome specific cleavage of the aminoluciferin substrates, in a parallel experiment the samples were incubated with the proteasome inhibitor epoxomicin for 1 h before measurement. Since no significant luminescence was detected in the presence of epoxomicin, the signals detected in the samples without epoxomicin were attributed to the activity of 20S proteasome.

### Cell Survival and Viability Assays

Strains were grown overnight in SD-His^−^ medium. Cultures were diluted to OD_600_ 0.15 in 6 ml of SD-His^−^ medium and incubated until ΔOD_600_ ≈ 1.0. Samples were collected in exponential phase or on days 3, 6, 9 and 14 (i.e., days after start of incubation). For the spot-assay, cells were pelleted, resuspended in water (to OD_600_ 0.2) and diluted in 10-fold series (10^−1^, 10^−2^, 10^−3^). 4.5 μl of each suspension was spotted on SD-His^−^ plates.

To assess the cell resistance to oxidative stress or heat shock, cells were incubated for 4 h in liquid SD-His^−^ medium supplemented with hydrogen peroxide at a final concentration of 20 mM (Sigma-Aldrich, Sweden) or incubated at 52°C for 2 h. Cell survival was analyzed by spot-assay after 3–4 days of incubation at 30°C.

Yeast cell viability was determined by staining with the fluorescent vital dye FUN-1 [2-chloro-4-(2,3-dihydro-3-methyl-(benzo-1,3-thiazol-2-yl)-methylidene)-1-phenylquinolinium iodide] (Life Technologies Europe BV, Sweden). Cells were harvested, washed and suspended in 10 mM HEPES buffer (2% glucose) with 15 μM FUN-1 and 25 μM Calcofluor White M2R (Life Technologies Europe BV, Sweden). Cells were then incubated at 30°C for 30 min protected from light before analysis by fluorescence microscopy using TL-DIC, FLUO-GFP, FLUO-RFP and FLUO-A4 settings. Metabolically active cells are able to transport and concentrate the dye in the vacuole where it forms structures in the shape of red bars or rods. Metabolically non-active cells give out a uniform glow, due to the non-specific distribution of the dye.

Every sample was measured in duplicate on two independent transformants and in three independent experiments.

### Inducing Protein Misfolding With L-Azetidine-2-Carboxylic Acid

L-azetidine-2-carboxylic acid (AZE, Aze or AZC) is a proline analog, that is incorporated into the proteins during translation, causing changes in protein conformation, and eventually misfolding (Zagari et al., 1990). Cells were grown overnight in SD-His^−^ medium, diluted to OD_600_ 0.15 in 6 ml and incubated at 30°C. At OD_600_ ≈ 1, AZE (Sigma-Aldrich, Sweden) was added to the culture to a final concentration of 30 mM. After 3 h of incubation, cells were pelleted and diluted to OD_600_ 0.2 in 1 ml of water. Serial dilutions (10^−1^, 10^−2^, 10^−3^) were used for spot-assays on SD-His^−^ plates. Growth was recorded after 2–3 days of incubation at 30°C.

For continuous treatment with AZE, cells were collected and diluted as before and spotted on SD-His^−^ plates containing 2, 4 and 7 mM AZE, respectively. Growth was recorded after 2–3 days of incubation at 30°C.

The viability was also measured by colony forming units (CFU). Cells were serially diluted in SD-His^−^ medium to 4 × 10^3^ cells/ml. One-hundred microliter of diluted cells was plated on SD-His^−^ plates, which were incubated at 30°C for 2–4 days. Viability of control strains was considered to be the initial survival (100%). Every sample was measured in duplicate on two independent transformants and in three independent experiments.

### Assessing the Apoptotic Markers

***The production of ROS*** was measured using dihydrorhodamine 123 (DHR123, Sigma-Aldrich, Sweden). Cells were collected at selected time points, washed and suspended in 50 mM sodium citrate buffer with (pH 5.0) 50 μM of DHR 123 and then incubated for 20 min at room temperature before analysis by fluorescent microscopy using YFP settings. Cells showing an intense fluorescence were treated as ROS accumulating cells.

***Cell membrane integrity*** was assessed by detecting loss of membrane asymmetry by exposed phosphatidylserine (PS), using Annexin V labeling (Life Technologies Europe BV, Sweden) and assessing membrane rupture by using propidium iodide (PI, Life Technologies Europe BV, Sweden). Cells were washed in sorbitol buffer (sorbitol 1.2 M, KH_2_PO_4_ 35 mM and MgCl_2_ 0.5 mM pH 6.8) and digested with 120 U/ml lyticase (Sigma-Aldrich, Sweden) for 2 h at 28°C (Madeo et al., 1997; Herker et al., 2004). After the incubation, cells were harvested by centrifugation (5000 rpm, 1 min), washed twice with 1 ml of cold PBS buffer (137 mM NaCl, 2.7 mM KCl, 10 mM Na_2_HPO_4_ and 1.76 mM KH_2_PO_4_, pH 7.4) and resuspended in Annexin-binding buffer (Life Technologies Europe BV, Sweden). Finally, 3 μl of Alexa Fluor 488 Annexin V and 1 μl of PI (100 μg/ml) were added to 100 μl of cell suspension and then incubated for 15 min at room temperature, protected from light. Finally the cells were centrifuged (5000 rpm, 1 min) and resuspended in 20 μl of Annexin-binding buffer for examination and analysis using a fluorescence microscope with FLUO-GFP and FLUO-RFP settings. Cells showing green fluorescence are apoptotic cells (PS externalization-positive), cells showing red fluorescence are necrotic cells (PI-positive) and cells showing fluorescence with both filters, are late apoptotic cells.

To analyze ***DNA breakage***, terminal deoxynucleotidyl transferase dUTP nick end labeling (TUNEL) assay was performed using the *in situ* Cell Death Detection kit (Roche AB, Sweden), as described previously (Madeo et al., 1999). Yeast cells were washed with PBS buffer, fixed with 3.7% formaldehyde and digested with 120 U/ml of lyticase for 40 min at 30°C. The cells were then harvested, washed with SPM buffer (sorbitol 1.2 M, KH_2_PO_4_ 50 mM and MgCl_2_ 1 mM pH 7.3) and transferred to a polylysine-coated slide. The slides were rinsed three times with PBS and incubated with permeabilization solution (0.1% sodium citrate, 0.1% Triton X-100) for 2 min at 4°C. After rinsing twice with PBS, 50 μl of TUNEL reaction mixture was added to the slide and incubated at 30°C for 60 min. For co-staining with DAPI (Life Technologies Europe BV, Sweden), 1 μl of DAPI (1 mg/ml) was added 5 min before the incubation time ended. Finally the slides were washed twice with PBS and mounting medium was added to analyze by fluorescence microscopy with FLUO-A4 and FLUO-GFP settings and DNA breakage could be visualized as fluorescence.

***In vivo detection of Caspase–like activity*** was done with 5 × 10^6^ cells taken on 6, 9 and 14 days of incubation, which were washed in PBS and resuspended in 200 μl of PBS containing 10 μM of a caspase substrate FITC-VAD-FMK (CaspACE FITC-VAD-FMK *in situ* marker, Promega Biotech, Sweden) and PI, to identify cells with ruptured plasma membrane indicating necrotic cell death, to a final concentration of 50 ng/ml. After incubation for 20 min (protected from light) at room temperature and with low agitation, cells were centrifuged and washed twice with PBS. Finally cells were centrifuged and resuspended in the remaining buffer. Stained cells were analyzed by fluorescence microscopy with FLUO-GFP and FLUO-RFP settings, and the bound marker is localized by fluorescence.

### Fluorescence Microscopy

Images from stained cells were taken on an inverted Leica AF 6000 fluorescence microscope (Wetzlar, Germany) with a HCX PL APO CS 100.0x1.40 OIL objective, captured with a DFC 360 FX camera, and analyzed with Leica Application Suite software. Quantitative determinations involved the analysis of at least 600 cells per sample from three independent experiments. Brightness and gain settings were adjusted to avoid background noise and discard false positives during the counting.

### Real-Time PCR Analysis of Chaperone Expression

UBB^+1^ expressing cells and control cells were grown in liquid selective medium at 30°C. Cell samples were taken on exponential phase (OD_600_ of 1.0), days 1 and 3 of incubation. Total RNA was extracted using the RNeasy^®^ kit (QIAGEN, Sweden) following manufacturer’s instructions. The synthesis of first strand cDNA was performed using 1 μg of total RNA and following the QuantiTec Reverse Transcription (QIAGEN, Sweden) manufacturer’s protocol. The primers were designed using the Primer3 software and synthesized by Sigma-Aldrich Company (Sigma-Aldrich, Sweden). The DyNAmo™ ColorFlash SYBR^®^ Green qPCR Kit (Thermo Fisher Scientific, Sweden) and the Mx3005P Agilent Technologies equipment (Agilent Technologies, Sweden) were used for the QPCR assay. *DAL81* snd *SPC10* were used as reference genes to normalize the RNA levels. The relative transcription levels were calculated using the ΔΔ*Ct* method. Results are from three independent experiments, performed duplicate per experiment. The data analysis was performed using the MX pro QPCR software.

### Statistical Analyses

Significance of differences observed between strains were determined using two-tailed, student *t*-tests. Unless specified explicitly, three independent replicates on two independent transformants were analyzed. Values were represented as mean ± SEM. *P* < 0.05 was considered to indicate significant difference.

## Results

### Expression of Human UBB^+1^ in Yeast *S. cerevisiae*

The DNA sequence coding for the human UBB^+1^ protein was codon-optimized for efficient expression in yeast *S. cerevisiae*. In order to ensure the transcription and translation of the correct mutated version, the GU nucleotides that are deleted during spontaneous and random molecular misreading of the native *UBB* gene in human cells, were omitted in the synthetic version, giving rise to the mutated *UBB*^+1^ gene (Figure 1A and Supplementary Figure S1). This *UBB*^+1^ gene was cloned into either the single- (centromeric, CEN) or multi-copy (2μ) yeast expression vectors, under the constitutive *TEF1* promoter, giving rise to p413TEF CEN UBB^+1^ and p423TEF 2μ UBB^+1^ plasmids, respectively (Mumberg et al., 1995), enabling its constitutive expression (Figure 1B). Yeast wild type strain CEN.PK 113-11C, a host for heterologous protein production (van Dijken et al., 2000), was transformed with the UBB^+1^ CEN or 2μ plasmids to generate the Low_UBB^+1^ and High_UBB^+1^ strains, respectively. Control strains (Low_EP and High_EP) were generated by transforming CEN.PK 113-11C with the empty CEN or 2μ plasmids (EP), without the *UBB*^+1^ gene. Transcription of *UBB*^+1^ was verified by RT-PCR (Figure 1C) and expression of UBB^+1^ protein was confirmed by Western blot (Figure 1D).

### Expression of UBB^+1^ Reduces the Proteolytic Activities of the 20S Proteasome

**Figure 2:**
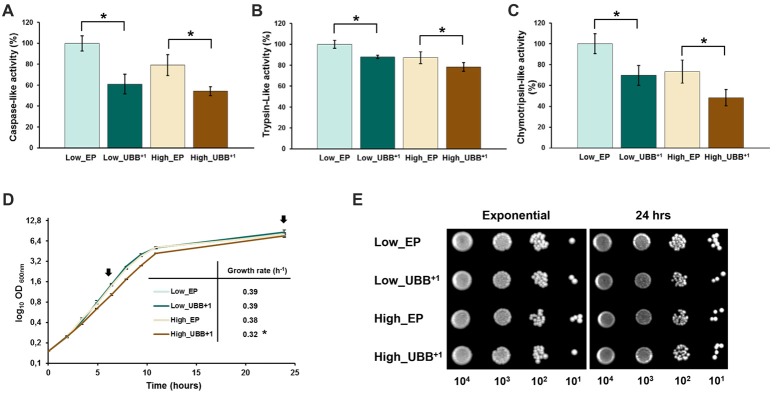
UBB^+1^ inhibits the proteolytic activities of the 20S proteasome and affects exponential growth. The luminescent assays were performed individually to measure the caspase-like, trypsin-like and chymotrypsin-like protease activities associated with the proteasome complex in cultured cells. Luminescence activity measurements of the **(A)** caspase-, **(B)** trypsin-and **(C)** chymotrypsin-like proteolytic activities of the 20S proteasome are shown. The data shown are average values of three independent experiments ± SEM. The asterisk (*) indicates significant differences (*p* < 0.05). UBB^+1^ strains were compared with its respective controls (Low_EP and High_EP). **(D)** The specific growth rate was determined from the maximal slope on logarithmic scale (μmax). The data represents an average of three independent experiments from two different colonies for each strain. Low UBB^+1^ expression did not affect cell growth while high UBB^+1^ expression slightly reduced growth rate (*p < 0.05). Arrows indicated the time points when samples were taken for spot survival assay **(E)** of control strains (Low_EP and High_EP) and UBB^+1^ expressing strains (Low_UBB^+1^ and High_UBB^+1^).

It has been previously shown that overexpression of UBB^+1^ reduces the activity of the UPS in HeLa and human neuroblastoma SH-SY5Y cell lines (Lindsten et al., 2002; Verhoef et al., 2009). We measured the three proteolytic activities of the 20S proteasome i.e., the caspase-like (β1), trypsin-like (β2) and chymotrypsin-like (β5) proteases activities in exponential Low_UBB^+1^ and High_UBB^+1^ strains, and corresponding controls. To this purpose luminogenic peptide substrates for the individual 20S proteolytic activities were used: Z-norleucine-proline-norleucine-aspartate-aminoluciferin, Z-leucine-arginine-arginine-aminoluciferin and Succinyl-leucine-leucine-valine-tyrosine-aminoluciferin for the caspase-like, trypsin-like and chymotrypsin-like activities, respectively. We found that both low and high UBB^+1^ expression caused a decrease in all three proteolytic activities of the proteasome (*p* < 0.05) when compared with its respective controls (Figures 2A–C). In the High_UBB^+1^ strain, the caspse-, trypsin- and chymotrypsin-like activities decreased up to 32%, 10% and 35%, respectively. The activities of these three proteases in Low_UBB^+1^ strain were reduced by 38%, 12% and 30%.

In order to evaluate the effects of a reduced proteasomal activity on cell growth due to UBB^+1^ expression, UBB^+1^ and control strains were cultured in liquid SD-His^−^ medium. Cell growth was followed during exponential phase and cell viability was measured in exponential and stationary phases by spotting on SD-His^−^ plates. Specific growth rate was determined for each strain from the maximal slope on logarithmic scale (μmax). Each experiment was conducted by triplicate on three independent experiments. While low UBB^+1^ expression did not affect exponential growth in liquid culture (*μ* = 0.39), the High_UBB^+1^ strain had a significantly reduced growth rate up to 16% (*μ* = 0.32) as compared to its control (*μ* = 0.38; Figure 2D). Moreover, cells expressing UBB^+1^ at lower level reached a higher cellular density after 1 day of incubation (OD_600_ 8.6 ± 0.2) as compared to controls (OD_600_ 7.9 ± 0.3) and the high_UBB^+1^ strain (OD_600_ 7.5 ± 0.25; Figure 2D). From the serial spot dilution assay, no difference on survival was observed on exponentially growing cells and 1 day old stationary cells expressing UBB^+1^ (Figure 2E).

### Low UBB^+1^ Expression Increases Cellular Tolerance to Chemically Induced Protein Misfolding

**Figure 3:**
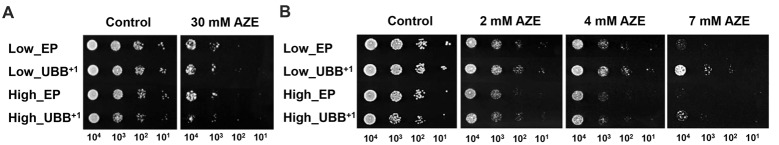
Low UBB^+1^ expression increases cellular tolerance to misfolded proteins. Spot tests to analyze the effect of chemically induced protein misfolding on survival of UBB^+1^ expressing strains and control strains. **(A)** Exponential cells, incubated for 3 h with 30 mM L-azetidine-2-carboxylic acid (AZE), were 10-fold serial diluted and spotted on selective plates. The control cultures were left untreated. **(B)** Exponential cells were spotted in 10-fold serial dilutions on plates containing 2 mM, 4 mM and 7 mM of AZE, respectively. Every sample was measured in duplicate on two independent transformants and in three independent experiments.

We investigated how exponentially growing cells, with a reduced proteasomal capacity, coped with misfolding burden, by generating misfolded proteins using amino acid analogs. The UPS is the main protein degradation pathway that eliminates misfolded or damaged proteins and its dysfunction may lead to protein accumulation, aggregation and neurodegeneration in mouse brain neurons (Bedford et al., 2008). To test the tolerance to protein misfolding stress, we challenged UBB^+1^ expressing cells with AZE. AZE is an analog of proline, whose structural difference results in changes in torsion angles, the direction of turns of the protein backbone, and cis-trans isomerizations in the random newly synthesized polypeptides that incorporate AZE instead of proline, inducing general misfolding stress (Tsai et al., 1990; Baeza et al., 2008). We incubated exponentially growing cells with 30 mM AZE for 3 h, then cells were diluted and spotted on standard plates. After 3 days of incubation, we observed that all strains were affected and no significant difference in survival was observed between UBB^+1^ strains and controls (Figure 3A). The number of viable cells was determined using CFU, which showed similar results to spot assay (Supplementary Figure S2A). We also observed a decreased viability in high_UBB^+1^ strain compared to its control strain (Supplementary Figure S2A).

A second experiment was conducted where cells were grown in liquid medium and spotted on plates containing different concentrations of AZE. Interestingly, the Low_UBB^+1^ strain showed better survival, as compared to controls and High_UBB^+1^ strain (Figure 3B and Supplementary Figure S2B). The expression of UBB^+1^ protein was also confirmed by Western blot during different concentrations of AZE treatment (Supplementary Figure S3). This shows that low UBB^+1^ expression, although inhibited the core proteolytical activities of the proteasome during exponential growth, increases the cellular capacity to cope with misfolded proteins during a prolonged misfolding stress.

### Low UBB^+1^ Expression Prolongs CLS of *S. cerevisiae*

**Figure 4:**
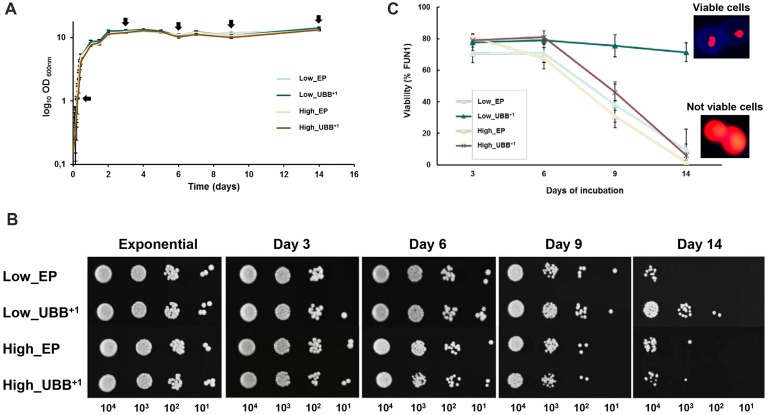
Low UBB^+1^ expression prolongs the CLS of *S. cerevisiae*. **(A)** Effect of UBB^+1^ expression on cell growth during chronological aging. All strains maintained similar biomass during stationary phase. Sampling for further analyses was performed at the indicated time points (black arrows). **(B)** Spot tests of Low_UBB^+1^, High_UBB^+1^ and control strains to investigate long time survival. 0.2 OD_600_ of cells were diluted in 10-fold series (10^−1^, 10^−2^, 10^−3^) and spotted on selective plates from the indicated days. **(C)** Percentage of cell viability was determined by FUN1 staining. Above 70% of the cells expressing Low_UBB^+1^ remained viable after 14 days of incubation. Representative images of metabolically viable cells and non-viable cells stained with FUN1 are shown. Viable cells showed concentrated red staining in vacuole while non-viable cells showed uniform red glow. Samples were measured in duplicate on two independent transformants and in three independent experiments.

Successful handling of misfolded proteins declines with age which thus contributes to protein accumulation and aggregation, a common feature of neurodegenerative diseases (Basaiawmoit and Rattan, 2010). We tested whether Low_UBB^+1^ cells could deal more efficiently with aging stress conditions. To evaluate the effect of UBB^+1^ expression during aging, cells were grown in liquid SD-His^−^ medium. Sampling was conducted in the exponential phase (OD_600_ of 1.0), and on days 3, 6, 9 and 14, after the inoculation (Figure 4A). Cell viability was assessed by taking identical amounts of cells (OD_600_ 0.2) to make serial dilutions and spot onto SD-His^−^ plates.

We found that Low_UBB^+1^ strain was much more viable after 14 days, when compared to controls and the High_UBB^+1^ strain (Figure 4B). This was supported by FUN1 staining, which is a vital dye that passively diffuses into the cytoplasm and gives a diffused green fluorescence. In the living cells the dye is transported to the vacuole where it creates compact foci with a striking red fluorescence, thus reducing the diffuse green cytoplasmic fluorescence. Formation of the fluorescent red cylindrical intravacuolar structures (CIVS) requires both plasma membrane integrity and metabolic activity. Controls and cells expressing UBB^+1^ at higher level showed a similar reduction in viability (*p* < 0.05) from day 9 as compared to the Low_UBB^+1^ strain (Figure 4C). The average viability for the Low_UBB^+1^ strain at day 14 was above 70%, while cells expressing higher level of UBB^+1^ and controls had the average viability below 10% (Figure 4C). During CLS, the expression of UBB^+1^ protein was confirmed by Western blot on day 3 and day 9 (Supplementary Figure S4).

### UBB^+1^ Prevents the Accumulation of ROS and Decreases Apoptotic and Necrotic Cell Death During Chronological Aging

**Figure 5:**
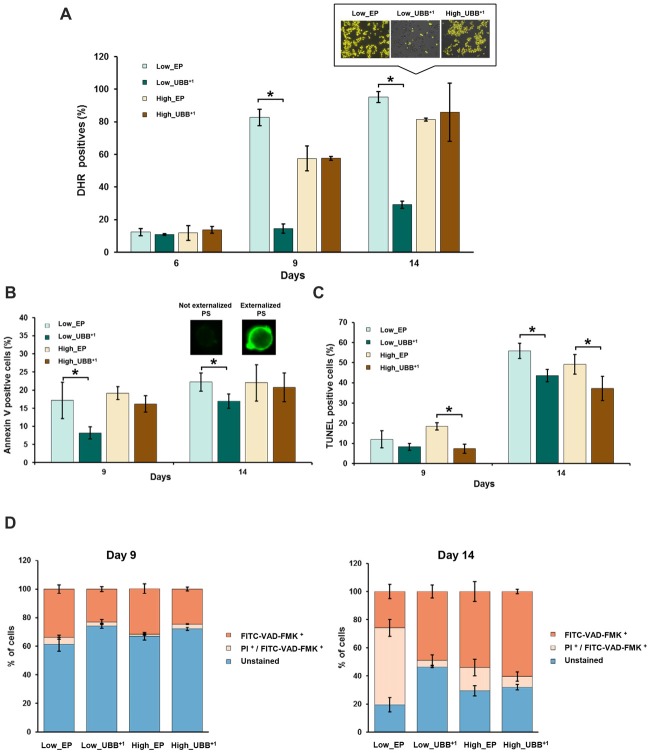
UBB^+1^ expression decreases cell death during chronological aging. **(A)** UBB^+1^ expressing cells and controls were stained with DHR 123 to determine the percentage of reactive oxygen species (ROS)-positive cells at different time points. Compared to control strain, low UBB^+1^ expression prevented cells to accumulate ROS at day 9 and day 14 (*p < 0.05). Representative images of DHR 123 stained cells from day 14 are shown. Cells showing an intense yellow fluorescence were counted as ROS accumulating cells. **(B)** Annexin V and **(C)** TUNEL assays were performed in UBB^+1^ expressing cells and controls to determine the percentages of cells exhibing phosphatidylserine (PS) externalization and DNA fragmentation after 9 and 14 days of incubation. UBB^+1^ expression, particularly at low levels, reduced the ocurrence of these apoptotic markers as compared to its control strain. **(D)** Stained cells with FITC-VAD-FMK and propidium iodide (PI) to determine the caspase-like activity and membrane integrity. Low levels of UBB^+1^ delays apoptosis on aged cells. After 14 days of incubation, controls showed a greater portion of cells in secondary necrotic stage or cells already dead as compared to low UBB^+1^ expressing cells, whose membrane integrity remains intact. The data shown were representative of average values ± SEM, of three independent experiments.

Deficiencies in either ubiquitin or proteasome can lead to the accumulation and aggregation of misfolded proteins with lethal consequences. It has been reported that proteasomal inhibition by UBB^+1^ overexpression contributes to proteotoxic stress, neuritic beading of mitochondria (Tan et al., 2007), cellular dysfunction and apoptotic cell death in HeLa and SH-SY5Y neuroblastoma cells (de Pril et al., 2004). In order to investigate the implications of UBB^+1^ expression in oxidative stress and cellular death, we measured the levels of intracellular ROS by using DHR123, where the nonfluorescent DHR is converted to the fluorescent product rhodamine 123 by the interaction with superoxides. ROS accumulated at very low levels during the first 6 days of incubation in all strains (between 11% and 13%; Figure 5A). As expected, controls showed an increased accumulation of ROS after 9 days of incubation since the generation of ROS is the first apoptotic trigger during chronological aging of yeast (Herker et al., 2004). Interestingly, a lower production of ROS was observed in Low_UBB^+1^ strain where the generation and accumulation of ROS were prevented after 9 and 14 days of incubation (Figure 5A).

Since ROS are the major inducers of apoptosis, we investigated whether lower ROS observed in the Low_UBB^+1^ strain correlated with reduced cell death. We focused on PS externalization, DNA fragmentation and the activation of caspase, as the markers of cell death. PS is actively maintained on the inner leaflet of the plasma membrane. In apoptotic cells externalization of the PS indicates the loss of plasma membrane asymmetry, which can be detected on the surface of the cell by Annexin V staining. After 9 days in culture, 17% of control cells and 16% of high UBB^+1^ expressing cells were positive in the Annexin V test, while only 8% of cells expressing low UBB^+1^ showed fluorescence (Figure 5B). After 14 days, the difference between strains was less prominent (Figure 5B).

DNA fragmentation is identified by the TUNEL assay, which is used for labeling DNA nick-ends (both single- and double-strand DNA breaks). In exponential phase and during first 6 days of incubation, all strains were negative in the TUNEL assay. After 9 days of incubation, 7%–8% of cells expressing UBB^+1^ showed fragmented DNA while slightly more cells (12%–18%) were positive in controls (Figure 5C). After 14 days of incubation, substantially more cells in controls exhibited DNA fragmentation (49%–56%) while 37% of cells expressing UBB^+1^ at high level were TUNEL positive, and 43% of UBB^+1^ at low expression displayed fragmented DNA (Figure 5C).

We finally measured the activation of caspases. In yeast, caspase-like activity contributes to apoptotic cell death under a variety of stimuli (Madeo et al., 2002, 2004), although caspase-independent apoptotic pathways have also been identified (Liang et al., 2008). It has been demonstrated previously, in human mononuclear cells from peripheral-blood that apoptotic cell death resulting from proteasome inhibition requires caspase activation in most cases (Pérez-Galán et al., 2006). To evaluate whether UBB^+1^ expression has an effect on caspase activation, we used FITC-VAD-FMK that can diffuse into the permeabilized cells and bind to activated caspases, allowing its identification by fluorescence microscopy. Co-staining with PI was performed to identify cells with ruptured plasma membrane, indicating necrotic cell death. Cells that are positive for the FITC-VAD-FMK assay alone, are considered induced apoptosis. Those that are positive in the PI assay are necrotic with ruptured plasma membrane and those positive in both assays, are late apoptotic. An increased number of cells showed caspase activation after 9 days, 32%–34% for the controls and 23% and 25% for Low_UBB^+1^ and High_UBB^+1^ strains, respectively (Figure 5D).

On 14-days old cells, the major caspase activity was measured in UBB^+1^ expressing cells (49% and 61% for low and high UBB^+1^ expressing cells, respectively) as compared to their respective controls (26% and 54%). However, co-staining with PI revealed that a large portion of control cells (17%–55%) stained for both FITC-VAD-FMK and PI while only 5%–7% of cells expressing UBB^+1^ were actually dead (Figure 5D). Additionally, and in accordance to our previous results on survival assay, a significant portion of cells expressing low level of UBB^+1^ remained unstained (46%) on day 14 (Figure 5D), demonstrating a beneficial effect on longevity.

### High UBB^+1^ Expression Increases Resistance to Heat Shock and Oxidative Stress

**Figure 6:**
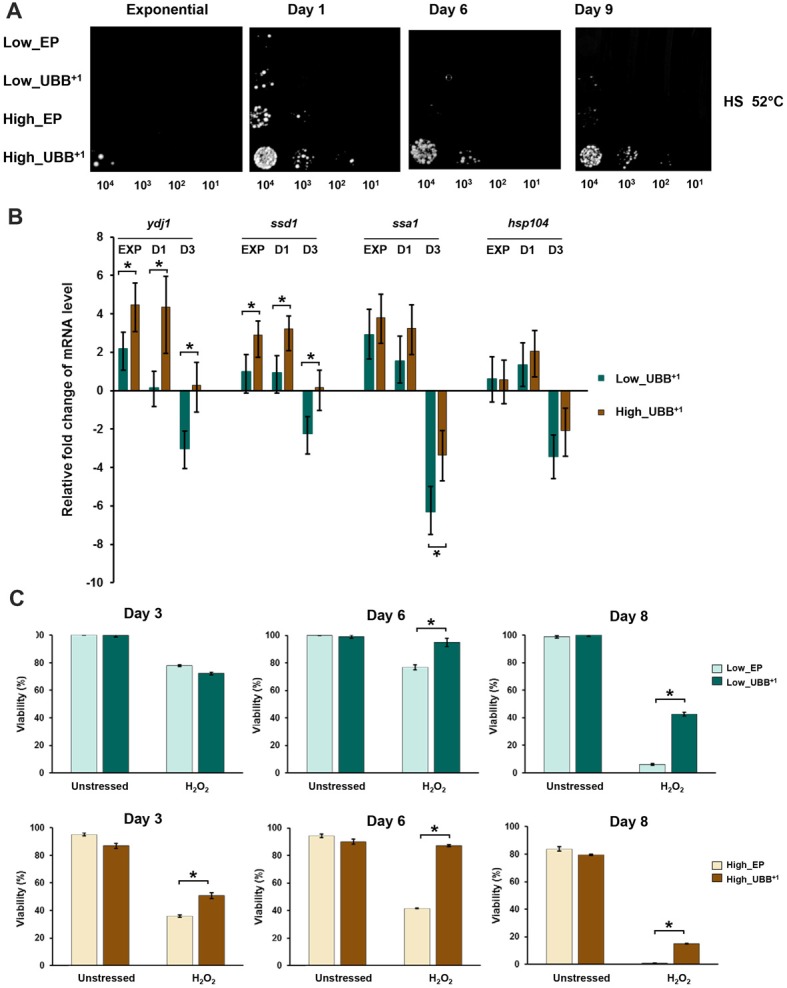
High UBB^+1^ expression increases yeast resistance to heat shock.** (A)** Spot assay of UBB^+1^ strains and control strains after heat shocked at 52°C for 2 h. Compared to other strains, high UBB^+1^ expression increased cell resistance to heat shock. **(B)** qPCR analysis of chaperone transcripts in low and high UBB^+1^ expression strains from exponential phase, day 1 and day 3. Both low and high levels of UBB^+1^ expression significantly increased the transcript levels of *Ydj1*, *Ssd1* and *Ssa1* genes in exponential phase and day 1. Comparing to Low_UBB^+1^ strain, the transcription levels of *Ydj1* and *Ssd1* were significantly higher in High_UBB^+1^ strain. The levels of these chaperone transcripts were significantly decreased after 3 days. **(C)** Viability of stationary cells exposed to 20 mM H_2_O_2_. Both of low and high UBB^+1^ expression increased cell resistance to oxidative stress. Results are average values ± SEM of biological triplicates. The asterisk (*) indicates significant differeces (*p* < 0.05).

A previous work in human neuroblastoma cells has reported the induction of heat-shock proteins caused by expression of UBB^+1^, which also promotes resistance to oxidative stress (Hope et al., 2003). In yeast cells, proteasomal inhibition confers thermotolerance due to the induction of chaperone expression and trehalose accumulation (Lee and Goldberg, 1998). We measured trehalose content in different strains under stress and control conditions and did not find significant differences (data not shown). We evaluated whether the proteasomal inhibition caused by UBB^+1^ induced a heat shock response protecting cells from stress. We evaluated the thermotolerance of UBB^+1^ expressing cells, in exponential phase (6 h) and on days 1, 6 and 9, respetively, by incubation of the liquid culture at 52°C for 2 h. 0.2 OD_600_ of cells were sampled, serially diluted and spotted on SD-His^−^ plates. After 3 days of incubation at 30°C, we observed that stationary cells (1, 6 and 9-days old cells) expressing UBB^+1^ at higher level were able to tolerate better to the heat stress, compared to controls and Low_UBB^+1^ cells (Figure 6A).

The transcription of chaperone genes (*Ydj1-Hsp40*, *Ssa1-Hsp70*, *Ssd1* and *Hsp104*) relevant for yeast thermotolerance was measured by real-time PCR. The trancription levels were compared between the UBB^+1^ expressing cells and its corresponding controls from samples taken from exponential phase (6 h), day 1 and 3, respectively. We observed that both low and high levels of UBB^+1^ expression significantly increased the transcript levels of *Ydj1*, *Ssd1* and *Ssa1* genes in exponential phase and day 1 (*p* < 0.05). Moreover, the transcription levels of *Ydj1* and *Ssd1* were significantly higher in the high UBB^+1^ expression strain, compared to low UBB^+1^ expresssion strain (*p* < 0.05). Interestingly, the levels of these chaperone transcripts were significantly decreased after 3 days (*p* < 0.05) and this was more pronounced in the low UBB^+1^ expressing strain (Figure 6B).

Since the induction of chaperones promoted resistance to oxidative stress, we exposed stationary cells (3, 6 and 8 days-old) to 20 mM H_2_O_2_. Better coping with oxidative stress was observed in both Low_UBB^+1^ and High_UBB^+1^ strains as compared to controls, particularly in aging cells (Figure 6C). There was no significant difference in exponentially growing cells (data not shown).

### Low UBB^+1^ Expression Increases Resistance to Misfolded Proteins in Autophagy Defective Cells

**Figure 7:**
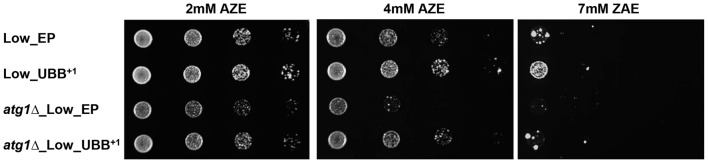
Exponentially growing Low UBB^+1^ expression resists to AZE-induced protein misfolding stress under autophagy defective background. Spot assay of Low_EP, Low_UBB^+1^, *atg1*Δ_Low_EP and *atg1Δ*_Low_UBB^+1^ cells on different concentrations of AZE. The expression of UBB^+1^ increased the cellular capacity to cope with misfolding protein stress in the absense of the autophagic pathway. Every sample was measured in duplicate on two independent transformants and in three independent experiments.

The UPS and autophagy machinery are two major protein degradation systems in cells. Several studies have suggested a functional cross-talk and it has been demonstrated that inhibition of UPS by proteasome inhibitors can activate autophagy. Autophagy induced by proteasome inhibition was shown to be important for controlling endoplasmic reticulum stress and reducing cell death in cancer cells (Ding et al., 2007). We tested if the autophagosomal pathway contributed to protect cells from toxicity under misfolding protein stress on cells expressing low level of UBB^+1^, where proteasomal activity was reduced. The *Atg1* gene, required for autophagosome, was deleted to generate cells defective in autophagy (unpublished data). The *atg1Δ* cells expressing UBB^+1^ and controls were grown in liquid medium and then diluted and spotted on plates containing 2, 4 and 7 mM of AZE, respectively. We observed a lower survival on cells defective in autophagy (*atg1Δ*) as compared to controls (Figure 7 and Supplementary Figure S5). Surprisingly, the expression of low UBB^+1^ increased the cellular capacity to cope with misfolded proteins on both WT and *atg1Δ* background, in 2 mM and 4 mM AZE (Figure 7). However, at the higher concentration of AZE tested (7 mM) only the UBB^+1^ strain (without the *Atg1* deletion) was able to thrive (Figure 7 and Supplementary Figure S5). This suggests that the autophagosomal pathway might be relevant for cell survival in some conditions but is not crucial in exponentially growing cells, during prolonged general misfolding stress induced by 4 mM AZE, when UBB^+1^ was present.

## Discussion

In this study we present a yeast UBB^+1^ model, with the ubiquitin mutant expressed constitutively at two levels. We used this system to explore the role of proteasome impairment and cell death by UBB^+1^ accumulation. It has been documented that the accumulation of UBB^+1^ protein is among hallmarks of several neurodegenerative diseases, including AD and other tauopathies (van Leeuwen et al., 1998b; Fischer et al., 2003). Studies in neuron cells have shown that UBB^+1^ can be further degraded by the proteasome, but it acts as a potent proteasomal inhibitor when it accumulates at high concentrations (Lam et al., 2000; Lindsten et al., 2002). Moreover, some reports showed that the proteasome activity is decreased in AD, reinforcing the idea that the accumulation of UBB^+1^ is related to proteasomal dysfunction (Keller et al., 2000). By measuring the proteolytic activities of the 20S proteasome in our yeast model, we demonstrated that expression of UBB^+1^, either at low or high levels, inhibited the caspase-, trypsin- and chymotrypsin-like proteases (Figures 2A–C). Although high UBB^+1^ expression caused a decrease in the growth rate of dividing cells, it did not affect cell survival of dividing cells, which reached similar biomass after 24 h in culture (Figures 2D,E).

Since proteasome dysfunction can lead to protein accumulation and aggregation with detrimental consequences, we evaluated the cellular tolerance to misfolded proteins in the presence of UBB^+1^. Surprisingly, low UBB^+1^ expression increased the tolerance to misfolded proteins when cells were challenged with AZE (Figure 3). Because neurodegenerative diseases, including AD and Huntington’s disease, are age-related disorders, we evaluated the effect of UBB^+1^ on chronologically aged cells and we found that low UBB^+1^ expression could extend yeast life span (Figure 4).

The impairment of proteosomal activity by UBB^+1^ overexpression has been shown to be detrimental to the neuronal cell cultures leading to mitochondrial stress (Tan et al., 2007) and triggering apoptosis (De Vrij et al., 2001). Based on our results, the constitutive low expression of UBB^+1^ in yeast decreased the levels of ROS (Figure 5A), which normally accumulates during aging. Consequently, UBB^+1^ expression caused a delay of apoptotic and necrotic cell death (Figure 5). High and induced expression of UBB^+1^ was found in different models to be toxic (Tank and True, 2009). Braun et al. showed that when UBB^+1^ was expressed from a high-copy vector under the control of an inducible promotor, the cytotoxicity (as shown by CFU, oxidative stress) occurred in the stationary phase (post-mitotic cells) and the toxic effects were observed 2 or 3 days after the induction (which was in exponential phase; Braun et al., 2015a). In our work, the expression of UBB^+1^ was constitutive which allowed the cells to adapt for example by overexpressing chaperones hence providing some cytoprotective functions. Our constitutive UBB^+1^ model seemed to produce enough of UBB^+1^ to cause some proteasomal inhibition, and a potentially protective response but not enough to be detrimental.

Hope et al. reported that in their human neuroblastoma cell model UBB^+1^ causes proteasome inhibition and induces the expression of HSPs, protecting cells against heat and oxidative stress conditions (Hope et al., 2003). Similarly in yeast, the exposure to proteasome inhibitors causes the expression of HSPs and the accumulation of trehalose conferring thermotolerance to *S. cerevisiae* (Lee and Goldberg, 1998). We demonstrated that high UBB^+1^ expression induced the transcription of HSPs in yeast and, in accordance to previous findings, increased the tolerance to oxidative stress (Figure 6). The induction of HSPs was observed in early time points (logarithmic phase and day 1), suggesting its activation as an initial protective mechanism to counteract with protein misfolding after stress. The up-regulation of Hsp104 and Ydj1 upon proteasomal inhibition and its involvement in increased resistance to high temperature of yeast is well documented (Lee and Goldberg, 1998). Our data showed a greater up-regulation of HSPs transcripts, particularly of *Ydj1*, in the presence of high levels of UBB^+1^ and consequently an increased resistance to heat shock in that strain (Figure 6B). Although trehalose has been reported to accumulate upon proteasomal inhibition having additive effects in causing thermotolerance, we did not observe any difference in the cellular content of this disaccharide upon UBB^+1^ expression (data not shown). Based on our test with the *Atg1* deletion mutant, we proposed that autophagy is not a crucial pathway for UBB^+1^ mediated survival during AZE-induced misfolding stress in exponentially growing cells.

The cell resistance to several stresses such as protein misfolding, oxidative stress and heat, induced by differential expression of UBB^+1^, is most probably not just due to the induction of chaperones or the autophagosomal pathway, but also to the participation of other cytoprotective mechanisms. In yeast cells (Tank and True, 2009) has shown that blocking ubiquitination of Ub^ext^ (a protein analogous to UBB^+1^) or weakening its interactions with other ubiquitin-processing proteins reduces the UPS impairment. Therefore, the interaction of Ub^ext^ with more than one protein is crucial to elicit impairment of the UPS (Tank and True, 2009). Another study showed that the accumulation of UBB^+1^ may be due to the inefficient hydrolysis of the C-terminus of UBB^+1^ by the UCHL3 under oxidative stress in AD (Dennissen et al., 2011). UBB^+1^ has been implicated to mediate neurodegeneration via interaction with other components of the UPS (Ko et al., 2010). The UBB^+1^ can serve as an inhibitor of DUB by binding to the Ubp6, the primary enzymes responsible for the disassembly of Lys48 linkages at proteasome (Krutauz et al., 2014). The UBB^+1^ can also be ubiquitylated by unusual ubiquitin-conjugating enzyme E2-25K and E3 ligating enzyme TRIP12 and HUWE1. The interation between E2-25K and UBB^+1^ is critical for the accumulation of UBB^+1^-anchored polyubiquitin, which results in proteasome inhibition and cell death (Ko et al., 2010).

We proposed that our UBB^+1^ yeast model might benefit to elucidate the contribution of different factors that together with UBB^+1^ may contribute to the disease initiation and/or progression (Supplementary Figure S6). Conservation of cellular protein quality control systems between yeast and human cells, makes yeast a useful model to study misfolding diseases. Humanized yeast models have been exploited to investigate pathological proteins and pathways for example, in Huntington’s disease (Giorgini et al., 2005), Parkinson’s disease (Outeiro and Lindquist, 2003) and AD (Chen et al., 2017). It is possible that the yeast discovery and validation platforms will provide new knowledge and tools for the identification of therapeutic strategies against neurodegenerative or other misfolding diseases (Chung et al., 2013; Tardiff et al., 2013).

## Author Contributions

AJM-A, AM, XC, EM and DP designed the experiments. AJM-A, AM, EM and XC performed the experiments. AJM-A, AM, XC and DP analyzed the data and wrote the manuscript.

## Conflict of Interest Statement

The authors declare that the research was conducted in the absence of any commercial or financial relationships that could be construed as a potential conflict of interest.

## Supplementary Material

FIGURE S1 Alignment of ubiquitin RNA sequences among human (Ubiquitin B *H.s*.), yeast (Ubiquitin *S.c*.) and *UBB*^+1^ codon optimized for expression in yeast.

FIGURE S2 CFU measurement of cellular survival under different concentrations of AZE treatment. **(A)** Exponential cells were incubated with 30 mM AZE for 3 h and plated on SD-His^−^ plates. **(B)** Exponential cells were plated on SD-His^−^ plates containing 2 mM, 4 mM and 7 mM of AZE, respectively. Results are average values ± SEM of biological triplicates.

FIGURE S3 Western blot analysis of UBB^+1^ expression under different concentrations of AZE treatment. GAPDH was used as a loading control.

FIGURE S4 Western blot analysis of UBB^+1^ expression during CLS on day 3 and day 9. GAPDH was used as a loading control.

FIGURE S5 CFU measurement of cellular survival under different concentrations of AZE treatment. Exponential cells were plated on SD-His^−^ plates containing 2 mM, 4 mM and 7 mM of AZE, respectively. Results are average values ± SEM of biological triplicates.

FIGURE S6 **(A)** Construction of the strains using a one copy or multicopy plasmid for the expression of the codon optimized UBB^+1^ sequence. **(B)** Evaluation of the strains on exponential phase. **(C)** Evaluation of the strains on stationary phase. Viability and the ocurrence of cell death markers were measured at the indicated time points. **(D)** Evaluations of the strains under stress conditions. Stress was induced either in liquid medium or on agar plates. The percentages shown are the results by comparing the UBB^+1^ strains with their respective controls.

## Abbreviations

AD: Alzheimer’s disease
AZE: L-azetidine-2-carboxylic acid
CLS: chronological life span
DHR123: dihydrorhodamine 123
HSPs: heat shock proteins
PI: propidium iodide
PS: phosphatidylserine
ROS: reactive oxygen species
TUNEL: terminal deoxynucleotidyl transferase dUTP nick end labeling
UPS: ubiquitin proteasome system.
