# Supplementary material for: Different Expression Levels of Human Mutant Ubiquitin B+1 (UBB+1) Can Modify Chronological Lifespan or Stress Resistance of Saccharomyces cerevisiae
Source: Front Mol Neurosci. 2018 Jun 8;11:200. doi: 10.3389/fnmol.2018.00200 (PMC6008557; doi:10.3389/fnmol.2018.00200)
Supplement: FIGURE S1 — Alignment of ubiquitin RNA sequences among human (Ubiquitin B H.s.), yeast (Ubiquitin S.c.) and UBB+1 codon optimized for expression in yeast. [file Presentation_1.PDF]

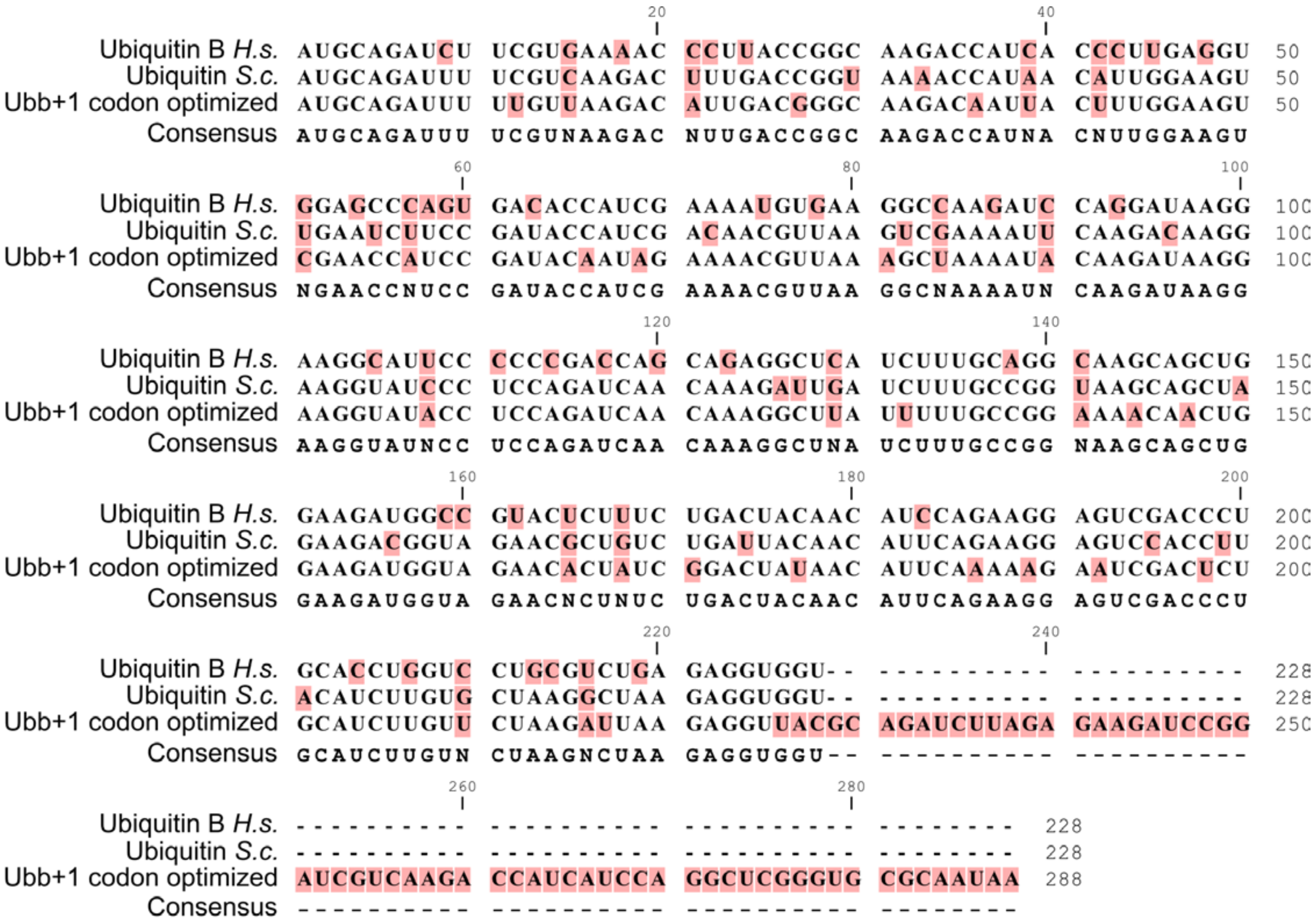

Fig. S1. Alignment of ubiquitin RNA sequences among human (Ubiquitin B *H.s.*), yeast (Ubiquitin *S.c.*) and Ubb^{+1} codon optimized for expression in yeast.

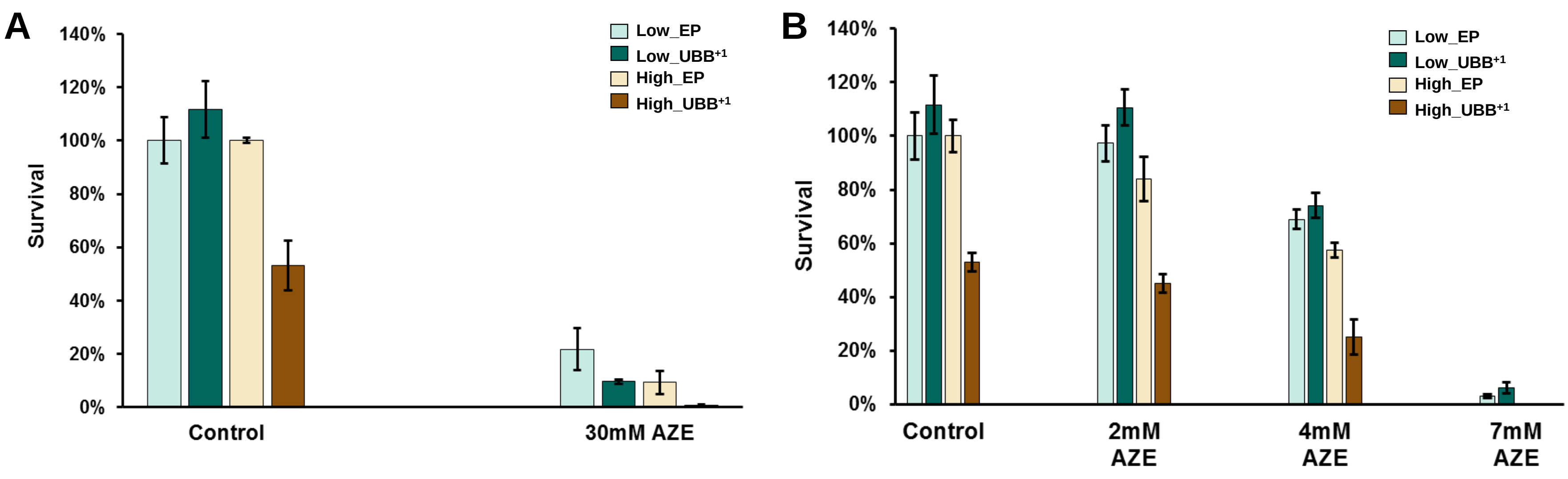

Fig. S2. CFU measurement of cellular survival under different concentrations of AZE treatment.

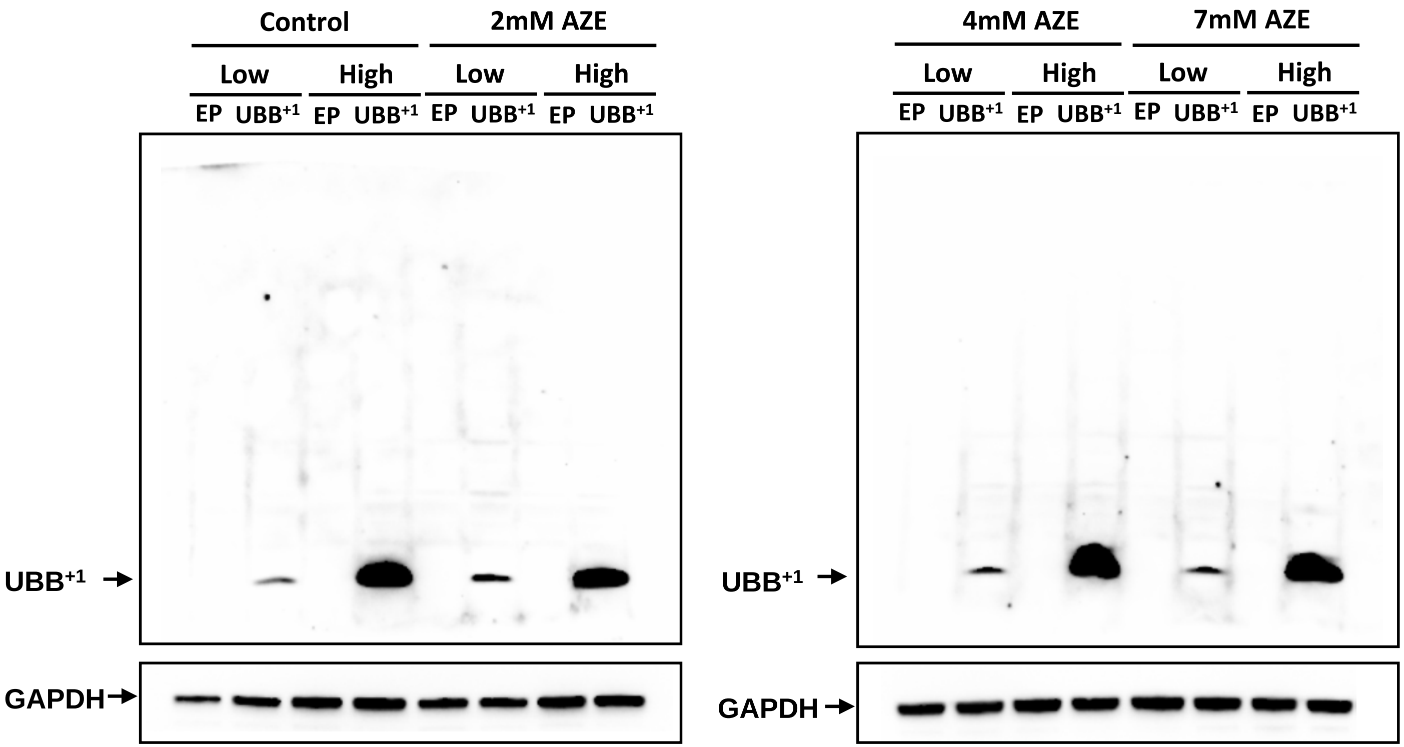

Fig. S3. Western blot analysis of UBB^{+1} expression under different concentrations of AZE treatment.

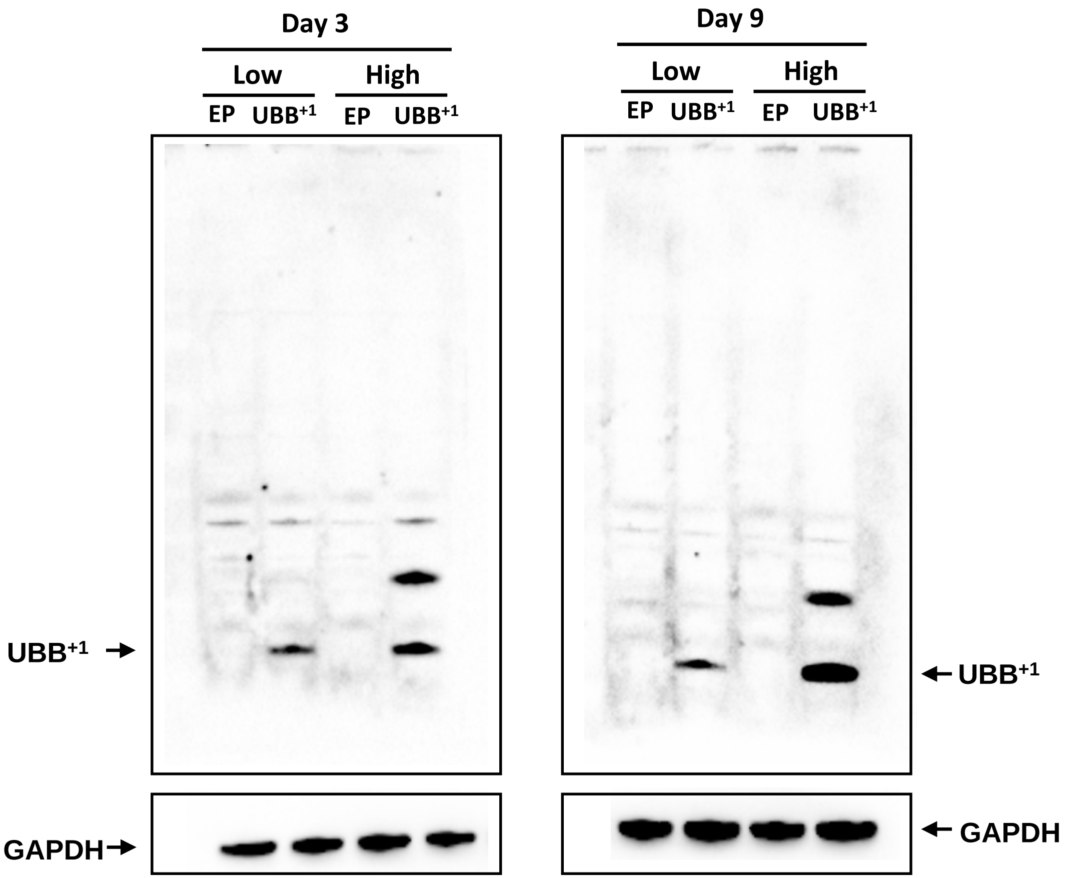

Fig. S4. Western blot analysis of UBB^{+1} expression during CLS on day 3 and day9.

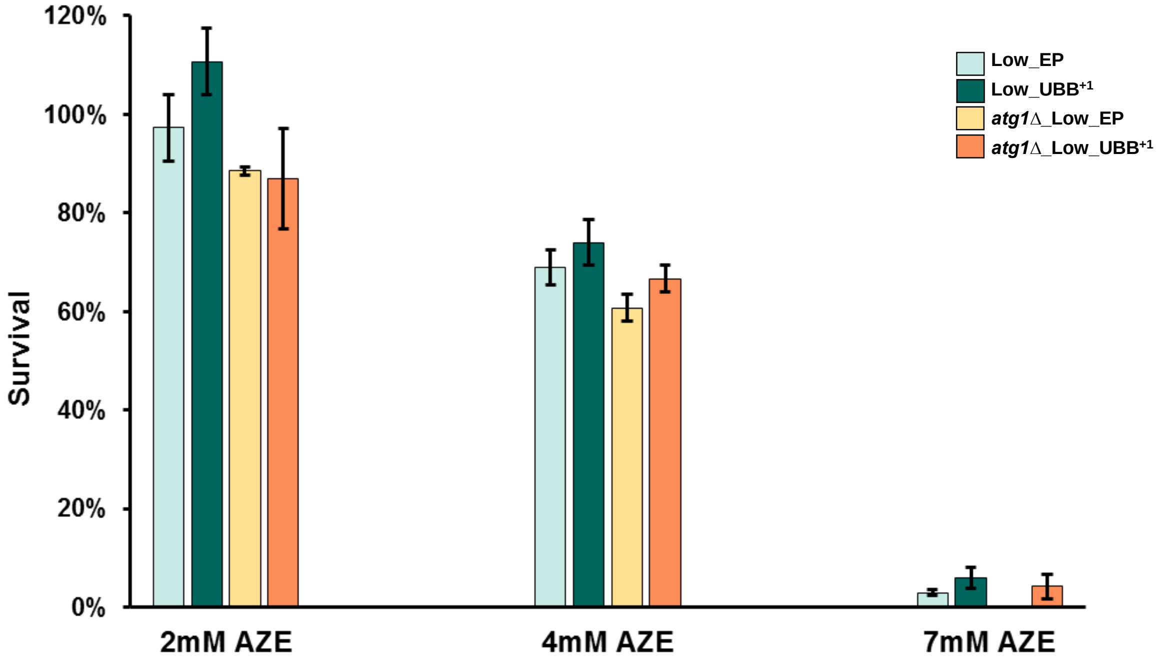

Fig. S5. CFU measurement of cellular survival under different concentrations of AZE treatment.

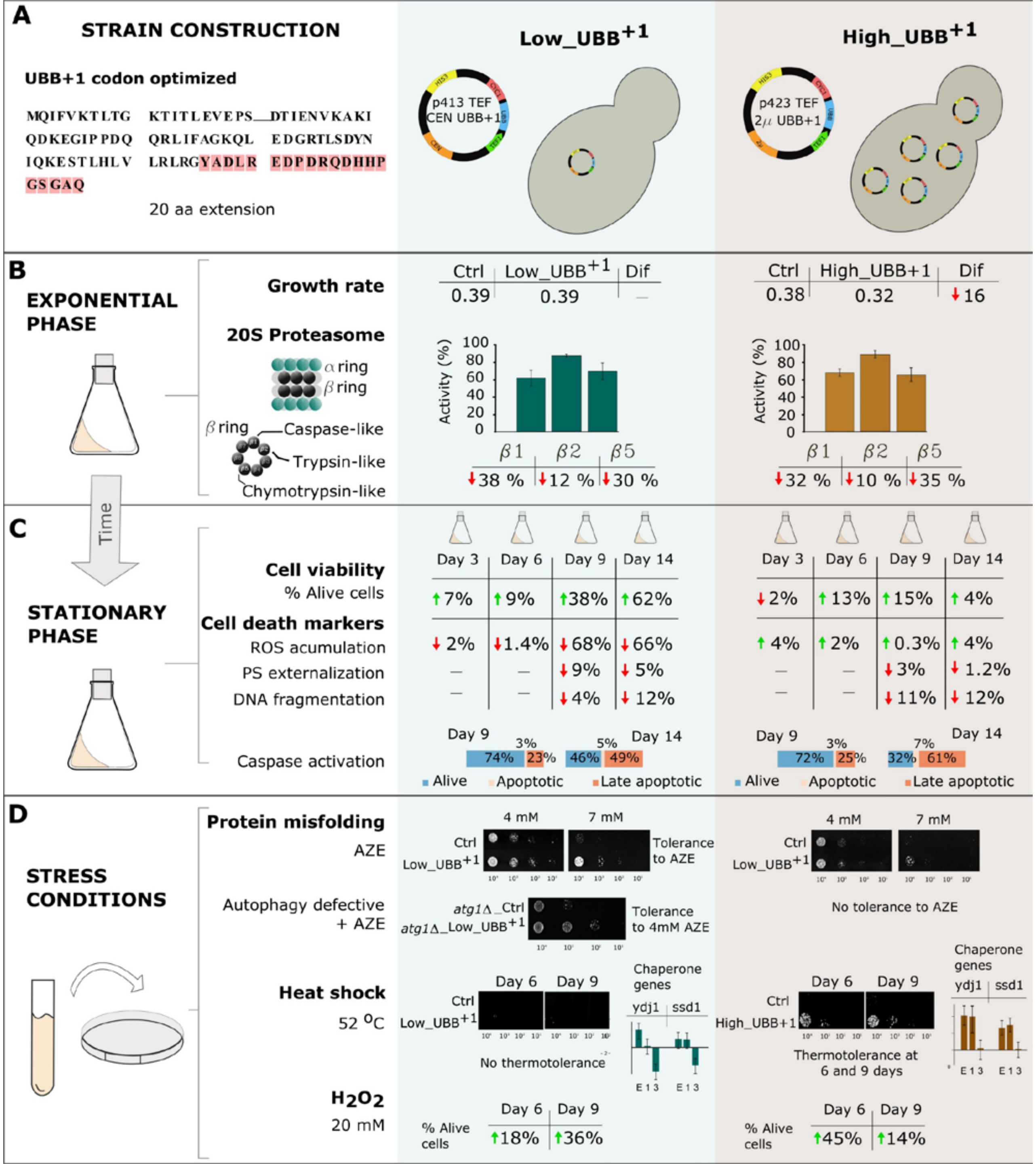

A
STRAIN CONSTRUCTION
UBB+1 codon optimized
MQIFVKTLTG KTITLEVEPS DTIENVKAKI
QDKEGIPPDQ QRLIFAGKQL EDGRTLSDYN
IQKESTLHLV LRLRGYADLR EDPDRQDHHP
GSGAQ
20 aa extension
Low_UBB+1
p413 TEF CEN UBB+1
High_UBB+1
p423 TEF 2μ UBB+1
B
EXPONENTIAL PHASE
Growth rate
Ctrl | Low_UBB+1 | Dif
0.39 | 0.39 | —
Ctrl | High_UBB+1 | Dif
0.38 | 0.32 | ↓16
20S Proteasome
α ring
β ring
β ring
Caspase-like
Trypsin-like
Chymotrypsin-like
Activity (%)
β1 β2 β5
↓38 % ↓12 % ↓30 %
↓32 % ↓10 % ↓35 %
Time
C
STATIONARY PHASE
Cell viability
% Alive cells
Cell death markers
ROS acumulation
PS externalization
DNA fragmentation
Day 3 Day 6 Day 9 Day 14
↑7% ↑9% ↑38% ↑62%
↓2% ↓1.4% ↓68% ↓66%
— — ↓9% ↓5%
— — ↓4% ↓12%
Day 3 Day 6 Day 9 Day 14
↓2% ↑13% ↑15% ↑4%
↑4% ↑2% ↑0.3% ↑4%
— — ↓3% ↓1.2%
— — ↓11% ↓12%
Caspase activation
Day 9: 74% 3% 23%
Day 14: 46% 5% 49%
Day 9: 72% 3% 25%
Day 14: 32% 7% 61%
Alive Apoptotic Late apoptotic
D
STRESS CONDITIONS
Protein misfolding
AZE
4 mM 7 mM
Ctrl
Low_UBB+1
Tolerance to AZE
No tolerance to AZE
Autophagy defective + AZE
atg1Δ_Ctrl
atg1Δ_Low_UBB+1
Tolerance to 4mM AZE
Heat shock
52 °C
Day 6 Day 9
High_UBB+1
No thermotolerance
Thermotolerance at 6 and 9 days
Chaperone genes
ydj1 ssd1
H2O2
20 mM
% Alive cells
Day 6 Day 9
↑18% ↑36%
↑45% ↑14%

Fig. S6
